# Sensor-Driven Human-Robot Synergy: A Systems Engineering Approach

**DOI:** 10.3390/s23010021

**Published:** 2022-12-20

**Authors:** Naoum Tsolakis, Antonios Gasteratos

**Affiliations:** 1 Department of Supply Chain Management, International Hellenic University, 570 01 Thessaloniki, Greece; 2 Institute for Bio-Economy and Agri-Technology (IBO), Centre of Research and Technology-Hellas (CERTH), 6th km Charilaou-Thermi Rd., 570 01 Thessaloniki, Greece; 3 Department of Production and Management Engineering, Democritus University of Thrace, Vas. Sophias 12, 671 32 Xanthi, Greece

**Keywords:** human–robot synergy, human–robot collaboration, human-centric agriculture, data and information flows, systems engineering, decision structure matrix

## Abstract

Knowledge-based synergistic automation is a potential intermediate option between the opposite extremes of manual and fully automated robotic labor in agriculture. Disruptive information and communication technologies (ICT) and sophisticated solutions for human-robot interaction (HRI) endow a skilled farmer with enhanced capabilities to perform agricultural tasks more efficiently and productively. This research aspires to apply systems engineering principles to assess the design of a conceptual human-robot synergistic platform enabled by a sensor-driven ICT sub-system. In particular, this paper firstly presents an overview of a use case, including a human-robot synergistic platform comprising a drone, a mobile platform, and wearable equipment. The technology framework constitutes a paradigm of human-centric worker-robot logistics synergy for high-value crops, which is applicable in operational environments of outdoor in-field harvesting and handling operations. Except for the physical sub-system, the ICT sub-system of the robotic framework consists of an extended sensor network for enabling data acquisition to extract the context (e.g., worker’s status, environment awareness) and plan and schedule the robotic agents of the framework. Secondly, this research explicitly presents the underpinning Design Structure Matrix (DSM) that systematically captures the interrelations between the sensors in the platform and data/information signals for enabling synergistic operations. The employed Systems Engineering approach provides a comprehensible analysis of the baseline structure existing in the examined human–robot synergy platform. In particular, the applied DSM allows for understanding and synthesizing a sensor sub-system’s architecture and enriching its efficacy by informing targeted interventions and reconfiguring the developed robotic solution modules depending on the required farming tasks at an orchard. Human-centric solutions for the agrarian sector demand careful study of the features that the particular agri-field possesses; thus, the insight DSM provides to system designers can turn out to be useful in the investigation of other similar data-driven applications.

## 1. Introduction

Harvesting and handling operations in high-value crops horticulture are labor-intensive, accounting, in many crops, for about 50% of the total production costs [1]. At the same time, agricultural worker availability is of great concern in many European countries [2] greatly vary in the labor productivity in the sector [3]. In addition, considering the share of employment in agriculture across the globe, it could be argued that agricultural labor productivity is lower compared to other industries [4]. Recently, mobility restrictions due to the COVID-19 pandemic resulted in additional disruptions in agricultural production, hence calling for innovative interventions to ensure resilience and sustainability in the agricultural value chain [5]. To this end, adopting digital technologies and the Internet of Things (IoT), such as automated machinery and agricultural robotics, offer innovative solutions contributing to the efficiency, sustainability, flexibility, agility, and resilience of agricultural supply chain operations [6]. However, replacing manual labor in agriculture production with fully automated machine-based systems, following the paradigm of the industrial manufacturing domain, has proven to require additional research to be effectively applied [7].

Most significantly, societal challenges that associate automation with unemployment growth typically hinder the adoption of digital technologies in rural communities [8]. Hence, knowledge-based synergistic automation as an intermediate option between the opposite extremes of manual and robotic labor could be a viable option [9]. Nevertheless, integrating automated mechanization in agricultural operations prior to maturing the technology could cause cost overruns and schedule deviations [10]. This observation is typical for electro-mechanical and software product and service platforms designed “*from the ground up*” without prior design knowledge [11]. A typical challenge of novel products and de novo designs refers to the poor analysis of the functional interactions between the modules of complex products [12]. However, the function of a product represents its operational purpose and is imperative to satisfy customer requirements [13] in order to ensure market success. In this respect, the product function in novel products is typically recognized at a late product development stage, resulting in time-consuming and costly interventions to avoid failures and ensure operational performance [14]. In addition, innovative technological systems may consist of multiple modules, each performing specific sub-functions and controlling a nexus of interrelations [15].

Human–robot interaction (HRI) systems comprise complex technology platforms representing endeavors that necessitate systematic conceptualization and designing for delivering the anticipated functionality and operational performance. The information and communication technology (ICT) modules (e.g., robotic fleet management, HRI for optimal logistics operations, and farmer activity recognition) have a crucial role in such knowledge-based platforms to ensure enhanced operational efficiency [16]. In such a synergy, human safety enabled via exploiting input information is apparently a prime factor, providing a harmonic coexistence of humans and robots [17,18].

In this regard, this research proposes a baseline human–robot synergy as the technology platform for agricultural operations in high-value crops. A human–robot synergy platform has to consist of: (i) The physical sub-system, i.e., a drone for mapping an agri-field and a mobile platform to execute the logistics operations; and (ii) the ICT sub-system for gathering data and managing data streams about the status of the agri-field and the farmer. This research especially analyzes the information sub-system of a human–robot synergistic system consisting of an extended network of sensors for enabling data acquisition to extract the context (e.g., worker’s status and environment awareness) and planning and scheduling the action agents of the platform. In this context, this research attempts to address the following Research Queries.

- Research Query #1—What could be a viable option in agricultural operations between the extremes of manual work and fully automated machine-based systems in agriculture?
- Research Query #2—How can the transition from conceptualization to the technology development of a human–robot synergy information sub-system in agriculture be realized?

Considering the exposure of supply chains to severe disruptions and the need to use data-driven digital technologies for supply chain security, cost-competitive resilience, and sustainability [19], it is critical to respond to the aforementioned research questions. Responding to Research Query #1, this research conceptualizes a human–robot synergy system for harvesting high-value crops. To address Research Query #2, this study focuses on the ICT sub-system and adopts a system engineering perspective to scrutinize it. In particular, this study applies the Design Structure Matrix (DSM), a technique developed by Steward [20] that provides a chaste and handy matrix format representation of the intra-system interactions of a complex engineering system.

Considering the low technology readiness level of automated systems for outdoor farming activities, this research contributes to the Systems Engineering field by providing an overarching analysis of the structure underpinning the data-enabled processes in human–robot synergy platforms. Notably, in the same vein as Tekinerdogan and Verdouw [21], this research proposes a catalog of data acquisition components that can be reused in the broad context of Systems Engineering. This catalog may support the Systems Engineering life cycle process regarding human–robot synergy platforms and is exemplified using a well-defined technology system in agriculture.

The rest of this paper is organized as follows: Section 2 inserts the relevant research background. Section 3 outlines the materials and methods for conducting this study. Section 4 conceptualizes the envisioned human–robot synergy system and provides an analysis of the structure of the developed robotic platform in a matrix format, particularly in terms of the information sub-system. The findings regarding the developed DSM model are also discussed. Finally, Section 5 presents conclusions, academic and practical implications, limitations, and future research avenues.

## 2. Research Background

Due to the advent of Industry 4.0 and the digitalization of the economy, technology-based implementations underlined by hardware and software configurations have been outlined for a plethora of operations in diverse fields. Indicatively, fully autonomous unmanned aerial vehicles combined with real-time computer vision algorithms have been investigated for human detection in search and rescue missions [22]. Specifically, the use of mobile robots in agriculture has a racted the focus of interest in recent years, with the ultimate objective of developing technology-based solutions for supporting/executing agricultural operations that are typically labor-intensive, time-consuming, and resource-demanding [7]. Considering the nascent character of agricultural digitalization, a common technical lexicon for autonomous applications in agriculture is not yet adopted [23], hence creating readership confusion. Lately, a stream of research studies has focused on technical details and modeling aspects of autonomous machines in agriculture.

In terms of planning the operations of mobile robots in agricultural environments, Moysiadis et al. [23] reviewed and synthesized the pertinent literature and identified several planning attributes for mobile robots, classified across three main categories, namely: (i) high-level control-specific attributes, (ii) operation-specific attributes, and (iii) physical robot-specific attributes. However, outdoor agricultural environments are complex and characterized by dynamic environmental conditions, as opposed to indoor manufacturing spaces [24]. Therefore, in order to enable the navigation of autonomous ground vehicles and inform subsequent operations, several data gathering and analysis techniques are required, including, for example, remote sensing, agri-field mapping, computer vision, data-driven segmentation, and classification algorithms. Besides, Anagnostis et al. [25] considered and tested a deep learning algorithm (named U-net) to accurately identify and segment tree canopies in orchards based on images captured via drones. In the same vein, regarding the autonomous navigation of mobile robots in semi-deterministic agricultural environments, Katikaridis et al. [26] documented a systematic manner in using drones to support the route planning of mobile robots in a real-world orchard of walnuts. The study developed and applied machine learning and noise reduction techniques for accurately representing an agri-field and exploring alternative navigation scenarios for the mobile robot. Furthermore, recognizing the need to address the spatial and temporal variability in outdoor environments, spatial mapping using unmanned ground vehicles equipped with depth cameras and drones for capturing corresponding three-dimensional orthomosaics has been developed [27]. The fusion of the collected datasets then informed the precise robot localization and navigation. Lastly, a method for constructing the local environment map and a reactive path tracker to accurately detect the tree row-end in an orchard and safely maneuver from one row to the other has been proposed by exploiting extreme alterations in the statistical distribution of points acquired by a depth camera compared to the points inside the row [28]. Besides, the human-centric philosophy acknowledges the essential notion of growing productivity without pi ing robots against workers for dominance of one or the other. Instead, robots should intertwist with humans and perform as partners rather than rivals [29].

Regarding HRI, natural communication pathways are required to enable synergistic ecosystems, as opposed to the conventional communication options between humans and computers via devices such as a keyboard, mouse, joystick, and touch screen [30]. A key constituent in HRI systems is safety while an automated machine is navigating in congestive environments; hence, collision avoidance or detection is a fundamental requirement in human–robot synergy platforms [31]. To this effect, novel local path planning methods have been proposed in the literature, incorporating, for example, the support vector machines theory [32]. Furthermore, activity recognition of workers is essential in HRI to enable the so-called “*social-aware robot navigation*” [33] toward human-centric practices. In agriculture, depth cameras and machine learning have been investigated to recognize hand gestures and enable real-time HRI [34]. In particular, the Robot Operating System has been utilized to “translate” human gestures into commands for the safe navigation of a robot in an agri-field. To a greater extent, to overcome the computational challenges of state-of-the-art human pose estimation architectures, alternative vision-based logarithmic approaches have been applied for efficient human-computer interaction [35].

## 3. Materials and Methods

**Figure 1:**
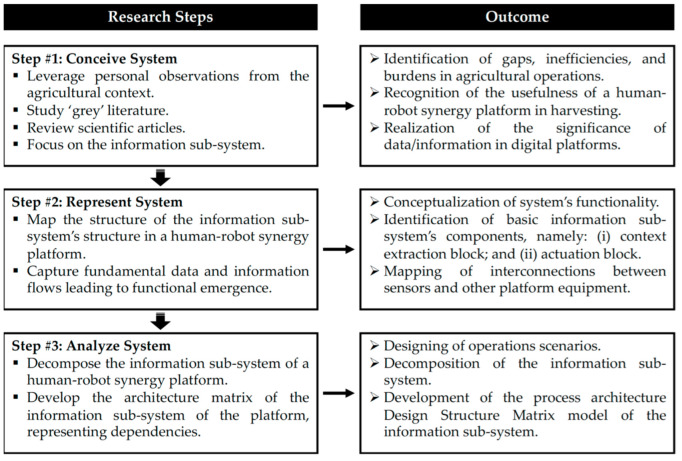
Research process flowchart.

Digital supply chain operations enabled by autonomous vehicles shall be analyzed based on an integrated multi-stage process involving conceptualization, simulation modeling, and deployment of testbeds in the real-world [36]. Embracing the system view of operations research, a challenging real-world issue has to be conceived, and a respective conceptual model has to be devised to comprehend the different governing parameters and variables and ultimately structure and delimit a viable solution [37]. To this end, this research first conceptualizes a human–robot synergy system, particularly focusing on the underpinning data and information flows. Then, a formalized method was adopted to analyze the system. The steps of the pursued research process followed in this study, along with the respective outcomes, are depicted in Figure 1.

### 3.1. Human-Robot Synergy Platform—Information Sub-System Structure

**Figure 2:**
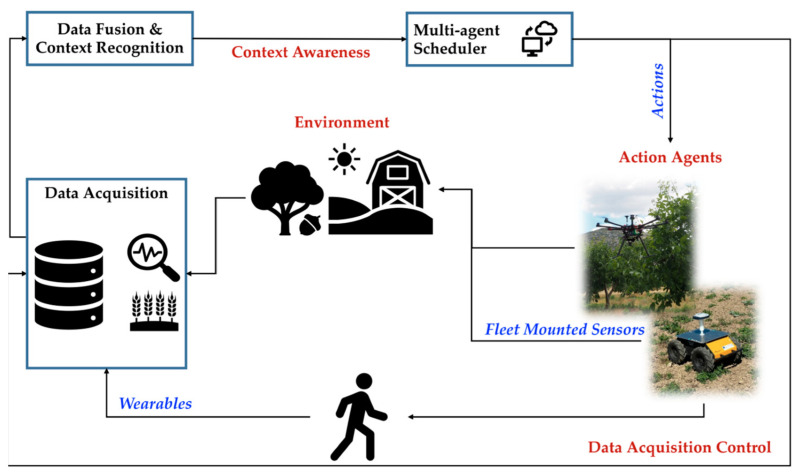
Graphical representation of the conceptual human–robot synergy system.

This research aims to denote the complexity of the information sub-system of the conceptualized human–robot synergy platform. The overall human–robot synergy system consists of: (i) The physical sub-system and (ii) the information sub-system (Figure 2). This research focuses on the information sub-system, mainly on the data gathering through the platform’s sensors and on its flows and processing to generate information for delivering the system’s function. In this regard, the information sub-system consists of the following:

#### 3.1.1. Context Extraction Block

In order to acquire data, sensors are installed at the user and action agents. Specifically, user agents bear wearable devices that perform continuous sensing independently of the agents’ attention and generate raw data. Overall, the data can refer to informed data, structured data, or the actual current context (e.g., the current activity of agents and the state of the surroundings). The human–robot synergy system, which this research explores, does not consider a network of sensory devices installed in the agricultural environment.

The context extraction block extracts context from the collected sensor data. Indicatively, during agricultural operations such as harvesting, the context can represent the worker’s current state (e.g., picking or loading a tray). In this regard, the wearables’ accelerometer data reveal the activity context of the worker.

#### 3.1.2. Actuation Block

The actuation block determines, prioritizes, and schedules actions for each executing agent in the system. Thus, if one agent in the field is blocked, the action scheduler plans an alternative route for the agent to follow so as to execute the task. This block also generates recommendations to user agents. For example, if one worker will be served in a delay, it recommends a rest period for this worker. Finally, this block also controls data acquisition; for example, if it detects a time period during which a worker exhibits low activity, then it reduces data acquisition load (i.e., switches off specific non-critical sensors) to save the energy on the worker’s wearable device.

### 3.2. Systems Engineering Analysis

**Figure 3:**
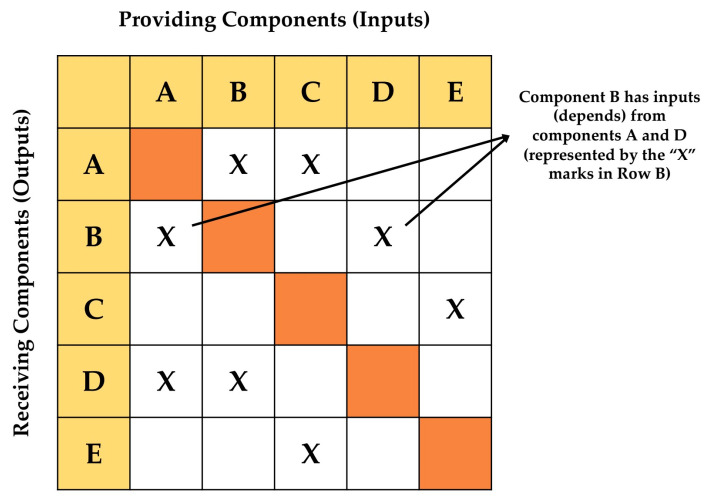
Simplified product/system architecture matrix representing dependencies (based on [39]).

The conceptual development of a human–robot information sub-system to support an integrated human–robot collaborative framework requires full documentation of the underlying process architecture, in particular the interrelations among the sub-system’s processes. To this end, this research applied DSM to map out the data interfaces of the information sub-system [38]. In principle, DSM maps the interactions among a set of *N* system elements as a square *N* × *N* matrix (Figure 3). This matrix represents the identified components comprising the system under investigation. The components’ interfaces are represented as marks in the matrix, which typically reveal patterns (modules or sub-systems) representing the system’s architecture. Specifically, the off-diagonal marks indicate the connections between a system’s elements.

The selection of DSM relies on the several benefits the technique offers [38], including:

1. Conciseness—The DSM’s structured arrangement of elements and their interactions in an *N* × *N* array offers a compact representation of a complex system.
2. Visualization—The DSM allows a designer to distinguish system modules or sub-systems of interest through congregating components and marking out regions of intense elements’ interactions, which in turn allow the indicative assignment of specific system components to a module to be deducted.
3. Comprehension—The DSM is easy to review and facilitates the understanding of the hierarchy and complexity of a system.
4. Analysis and Optimization Potential—The DSM allows power analyses and matrix mathematics to optimize a system’s structure, modularity, and other significant patterns.

## 4. Results

This section firstly presents the operations scenario for the conceptualized human–robot synergy platform as the object of inquiry and secondly analyses the design structure of the ICT sub-system.

### 4.1. Operations Scenario and System’s Functionality

Human–robot synergy in logistics operations for high-value crops requires the coordination and collaboration of the human and mechanized agents in the agri-field. Preferably, the environment should be semi-structured, e.g., orchards, to avoid encountering random and unexpected conditions that hinder the system’s functionality. In the conceptual system under consideration, the underpinning logic is to assist farmers in harvesting operations. Thus, the efficiency of the overall operation shall increase by minimizing a worker’s non-productive time, while the farmer’s well-being will be promoted by eliminating the required manual effort to carry heavy loads across the agri-field.

The manually operated drone, equipped with an RGB camera, performs flights over the agri-field of interest (preferably a structured orchard for ensuring stable environmental conditions to the overall portfolio of operations). The drone’s flights aim to map the orchard and help identify entities and any obstacles (e.g., trees and unknown objects like rocks). The acquired map is transmitted to a Farming Operating System (FMIS), where possible agri-field tracks for the routing of the mobile robot are extracted. The embedded algorithms then calculate the optimal path for the mobile robot.

Then, the mobile robot autonomously traverses the agri-field following the optimal route plan generated by the FMIS. The mission of the mobile robot is to safely approach the farmer, wait for a tray to be loaded onto the vehicle, transport the tray of high-value crops toward the exit of the farm, and then return to the next tree that the farmer serves until harvesting operations are completed (subject also to the generated permutation of the trees to serve in the optimal plan and the battery level of the mobile robot). For navigating within the agri-field, the autonomous vehicle is equipped with a Real-Time Kinematic GPS, while a LiDAR sensor also helps to avoid obstacles and recognize the status of the farmer.

Moreover, the human agent performs picking/harvesting operations of high-value crops (e.g., fruits). The farmer is equipped with five wearable sensors (viz., inertial measurement units—IMUs) that produce signals regarding the farmer’s body posture and movement. The signals are transmitted to the mobile robot for the automatic awareness of the situation and activity to ensure safety and facilitate interaction in an efficient and fenceless manner [40]. Specifically, the mobile robot can automatically identify the status of the farmer (e.g., work rate, task completion progress, abnormality detection) and react accordingly.

**Figure 4:**
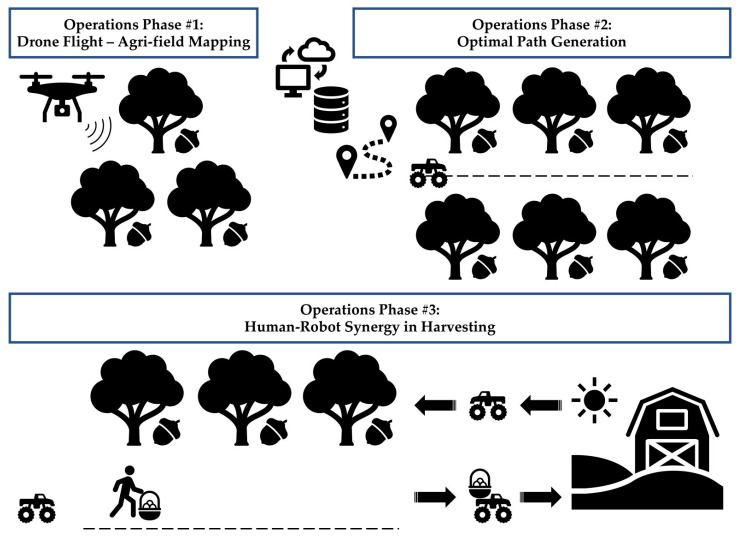
Operations scenario of the human–robot synergy system.

Last, to gather signals and develop “activity signatures” of potential farmers, experimental sessions of participants shall be organized [25]. The situation and activity recognition will be automatically registered in the FMIS. Notably, the experimental sessions would be required for every platform’s module and for the entire system, at both laboratory and actual agri-field environments, for validation, verification, and calibration purposes. The operations scenario that illustrates the functionality of the conceived human–robot synergy system is depicted in Figure 4.

### 4.2. Information Sub-System’s Decomposition

**Figure 5:**
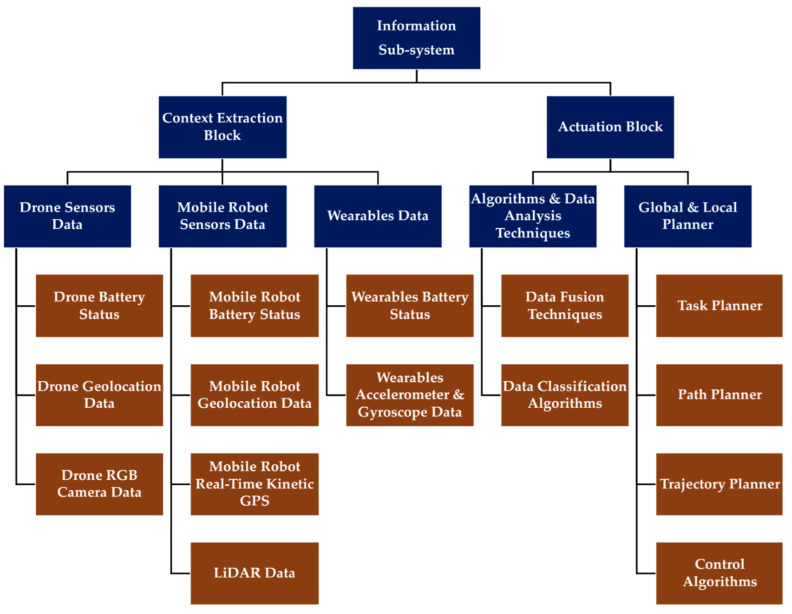
Decomposition of the information sub-system.

The information sub-system’s decomposition is presented in Figure 5. The resulting diagrammatic tree’s structure contains: (i) Form (objects); (ii) three levels of hierarchy, including level zero; and (iii) components, only regarding data acquisition and not the entire electro-mechanical system as this extends beyond the scope of this research.

**Figure 6:**
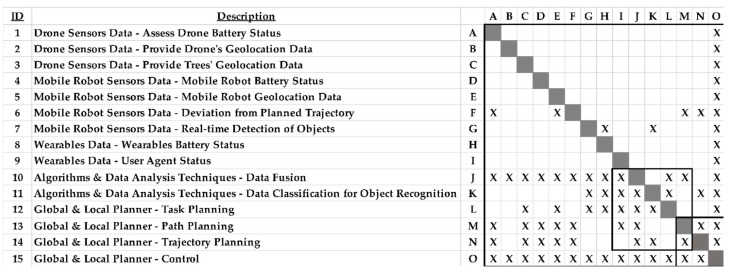
Process architecture DSM model of the information sub-system.

A process DSM is provided below (Figure 6) to identify tasks and respective data generated regarding:

- System tasks that can be executed in parallel.
- The sequence in which the system tasks have to be executed.
- System tasks, which have to be executed together due to any underlying dependencies.

### 4.3. Discussion

The process architecture DSM model in Figure 6 represents the information sub-system of a human-centric worker-robot logistics synergy for supporting harvesting in high-value crop agriculture. It is a 15 × 15 matrix, meaning that there are fifteen data-driven tasks performed based on the respective flows (indicated by a unique ID on the left-hand side of Figure 6). The data and data/information analyses and flows regard all the agents in the system, namely the drone, the mobile robot, and the user. The drone sensors provide data regarding the status of the flying platform itself and the geolocation of the trees in an agri-field. The mobile platform sensors generate data about the vehicle itself (as in the drone) and about the presence of any unexpected object in the agri-field to navigate safely. The wearables provide data about the status of the farmer that allow the mobile robot to act in a human-centric manner, e.g., approach the farmer or stop moving in advance to avoid an accident. The algorithms and the data analysis techniques permit data classification of the gathered information and enable context recognition in the agri-field. The global and local planners calculate the optimal paths for the agents to perform the pursued agricultural tasks.

Most of the marks (“X”) in the DSM model are below the diagonal hence representing feed-forward data flows. Along the diagonal, groups of data elements that interact are indicated. Two groups of data-driven tasks are executed together (i.e., ID 10–14 and ID 12–15, as marked out in Figure 6). These groups denote highly iterative groups of tasks and provide the designers with an indication of areas where the redesign is feasible. In context, planning the operations in diverse agri-field settings may require the selection of a different optimization objective (e.g., time, cost). To this effect, the DSM helps develop a taxonomy of optimization algorithms that would inform the planner. For example, the optimal routing of the mobile robot in the agri-field could be amenable to alternative criteria (e.g., formulate Eulerian path or Hamiltonian path) that could require alternative algorithmic approaches (e.g., Dijkstra’s algorithm or nearest neighbor search) (i.e., ID 10–14). The generated path would then be used as input to control the functionality of the physical counterpart of the platform.

In the developed process architecture DSM model of the information sub-system, the marks above the diagonal marks represent data flows that loop back and update other data/information silos and can reveal planned and unplanned iteration paths where improvements could be inserted for better system performance. Characteristically, all data and information are used to control the system’s behavior.

## 5. Conclusions

Harvesting of high-value crops (e.g., fruits and vegetables) is still performed manually in many agricultural settings and has been characterized by decreased growth rates in crop production in several countries. Considering urbanization as a social phenomenon and the migration of workers to urban centers, technological options can assist to achieve higher agricultural productivity. In this context, the main topic of this study is the seamless and fair work distribution between humans and robots through a human-centric approach to harvesting high-value crops as a response to the productivity challenges in agriculture.

Concerning the posed Research Query #1, this study conceptualized a human–robot synergy system as a technology platform for bridging the gap between the extremes of either manual or entirely automated agricultural activities. Besides, in response to Research Query #2, this research applied DSM to generate a matrix representation of the information sub-system of a baseline human–robot synergy system for high-value crops in agriculture. Installing a responsive information sub-system in a human–robot synergy platform is vital, as surveys revealed that collisions and human errors are the most common accidents in cases where humans and machines interact in contemporary farms.

### 5.1. Academic Contributions

The provided process architecture DSM model of the information sub-system can be used in an abstract manner to the early stage of designing a digital technology in different agricultural environments for performing diverse agricultural activities, hence contributing to the robotics fields [41]. Furthermore, in the digitalization discourse, the DSM model could act as a roadmap/template for designing context-aware human–robot synergistic operations enabled by sensing equipment, thus supporting the realization of “digital twins” in agriculture [42]. Overall, the contribution of this research to the Systems Engineering field can be encapsulated in the provided analysis of the baseline structure underpinning the data-enabled processes in human–robot synergy platforms.

### 5.2. Practical Implications

The provided analysis approach and the developed DSM model shall create insights for system engineers and managers on designing, organizing, implementing, and maintaining an ICT sub-system in human–robot synergy technological platforms. Specifically, the square grid representation helps unravel the interactions among data and information elements in human–robot synergy and could act as a useful tool for teams of engineers to technically align, introduce improvements and efficiently develop product modules and system functionalities that can be valuable to the end user.

### 5.3. Limitations

A few limitations characterize this study, which, however, provide interesting grounds for further research. Firstly, this research is limited only to the human–robot information sub-system, while the physical sub-system is not analyzed. Nonetheless, an integrated analysis shall consider both sub-systems as data and information exchange control the functionality of the human–robot synergistic system. Secondly, the provided analysis is qualitative in nature and is recommended at the design stage of a novel technology. Further validation requires the development and deployment of a real-world equivalent implementation to consider specific technical features.

### 5.4. Future Research

This research presented a human–robot synergy system by considering a set of sensors installed on all agents. However, recent research evidence documents the use of Machine Learning in replacing the need for sensor equipment, e.g., recognizing gestures to interact with the user instead of using wearables [34]. For example, research developments regarding IMUs and kinematic models of exoskeletons could be adopted and integrated with the system to enable more granular data gathering, hence allowing for more precise and efficient operations [43], specifically in agricultural settings. Such advancements would require updating the DSM as additional data and information flows are created.

Furthermore, this study develops a process architecture DSM model that represents the as-is sequence of data/information flows to control tasks in a human-centric robotic system. Conducting further analysis, as described in Eppinger [44], would be useful to navigate alternative sequences and/or definitions of data-driven tasks. Designing human-centric automated systems for agriculture requires consideration of the characteristics of the particular agri-field and crops where it would be applied, further reflecting upon the decisions pertinent to the downstream supply chain (e.g., supply-demand balance) [45].

## Data Availability

Not applicable.
